# Constructing Archival Geospatial Datasets to Link Historical Structural Racism to Cognitive & Functional Decline

**DOI:** 10.1093/geroni/igaf122.950

**Published:** 2025-12-31

**Authors:** Richard Sadler, Danielle Beatty Moody, Ayla Novruz, Allie Akmal, Rachel Davis, Graham Mooney, Kevin Henry, Robert Belli

**Affiliations:** Michigan State University, East Lansing, Michigan, United States; Rutgers University, New Brunswick, New Jersey, United States; University of Maryland, Baltimore County, Baltimore, Maryland, United States; Rutgers University, New Brunswick, New Jersey, United States; Rutgers University, New Brunswick, New Jersey, United States; Johns Hopkins University, Baltimore, Maryland, United States; Temple University, Philadelphia, Pennsylvania, United States; University of Nebraska-Lincoln, Lincoln, Nebraska, United States

## Abstract

More accurately measuring lifecourse environmental exposures requires working with data not readily available from digitized sources. Here we detail the processes for scouring city archives to uncover aspects of structural racism in the built environment. In our initial non-human subjects work in Baltimore, we have built datasets on the built environment and prepared methods to collect residential histories, which together allow us to study impacts of residential exposures across the lifecourse for this longitudinal cohort of aging adults. We discuss the iterative process of archive visits—working with team members who are experts in this field—to obtain the full complement of features in the built environment driven by structurally racist policies (i.e. redlining, blockbusting, restrictive covenants, urban renewal, freeway construction, predatory lending). We then discuss how residential histories are collected from our cohort, giving us lifecourse perspectives on where individuals have lived. These allow for the assignment of age- and period-specific exposures (e.g. one might expect developmental exposure to proximal neighborhood change to be more impactful than adult exposure to stable neighborhoods). These environmental data will eventually be spatially and temporally joined to our health data in GIS software, and multilevel models will allow us to examine what features of the environment and durations of exposure matter most to cognitive decline. Such data allow us to move ‘beyond redlining’ and other simplistic methods to consider more complex exposure frameworks.

